# Assessing left atrial intramyocardial fat infiltration from computerized tomography angiography in patients with atrial fibrillation

**DOI:** 10.1093/europace/euad351

**Published:** 2023-11-27

**Authors:** Andrea Saglietto, Giulio Falasconi, David Soto-Iglesias, Pietro Francia, Diego Penela, José Alderete, Daniel Viveros, Aldo Francisco Bellido, Paula Franco-Ocaña, Fatima Zaraket, Darío Turturiello, Julio Marti-Almor, Antonio Berruezo

**Affiliations:** Arrhythmia Department, Teknon Heart Institute, Teknon Medical Center, C/Vilana 12, 08022 Barcelona, Spain; Division of Cardiology, Cardiovascular and Thoracic Department, ‘Citta della Salute e della Scienza’ Hospital, Turin, Italy; Department of Medical Sciences, University of Turin, Turin, Italy; Arrhythmia Department, Teknon Heart Institute, Teknon Medical Center, C/Vilana 12, 08022 Barcelona, Spain; IRCCS Humanitas Research Hospital, Electrophysiology Department, Rozzano, Milan, Italy; Campus Clínic, University of Barcelona, C/Villarroel 170, Barcelona, 08024, Spain; Arrhythmia Department, Teknon Heart Institute, Teknon Medical Center, C/Vilana 12, 08022 Barcelona, Spain; Arrhythmia Department, Teknon Heart Institute, Teknon Medical Center, C/Vilana 12, 08022 Barcelona, Spain; Department of Clinical and Molecular Medicine, Cardiology Unit, Sant’Andrea Hospital, University Sapienza, Rome, Italy; Arrhythmia Department, Teknon Heart Institute, Teknon Medical Center, C/Vilana 12, 08022 Barcelona, Spain; IRCCS Humanitas Research Hospital, Electrophysiology Department, Rozzano, Milan, Italy; Arrhythmia Department, Teknon Heart Institute, Teknon Medical Center, C/Vilana 12, 08022 Barcelona, Spain; OpenHeart Foundation, Barcelona, Spain; Arrhythmia Department, Teknon Heart Institute, Teknon Medical Center, C/Vilana 12, 08022 Barcelona, Spain; Arrhythmia Department, Teknon Heart Institute, Teknon Medical Center, C/Vilana 12, 08022 Barcelona, Spain; OpenHeart Foundation, Barcelona, Spain; Arrhythmia Department, Teknon Heart Institute, Teknon Medical Center, C/Vilana 12, 08022 Barcelona, Spain; Arrhythmia Department, Teknon Heart Institute, Teknon Medical Center, C/Vilana 12, 08022 Barcelona, Spain; Arrhythmia Department, Teknon Heart Institute, Teknon Medical Center, C/Vilana 12, 08022 Barcelona, Spain; OpenHeart Foundation, Barcelona, Spain; Arrhythmia Department, Teknon Heart Institute, Teknon Medical Center, C/Vilana 12, 08022 Barcelona, Spain; Arrhythmia Department, Teknon Heart Institute, Teknon Medical Center, C/Vilana 12, 08022 Barcelona, Spain

**Keywords:** Atrial fibrillation, Adipose tissue, Intramyocardial infiltration

## Abstract

**Aims:**

Epicardial adipose tissue might promote atrial fibrillation (AF) in several ways, including infiltrating the underlying atrial myocardium. However, the role of this potential mechanism has been poorly investigated. The aim of this study is to evaluate the presence of left atrial (LA) infiltrated adipose tissue (inFAT) by analysing multi-detector computer tomography (MDCT)-derived three-dimensional (3D) fat infiltration maps and to compare the extent of LA inFAT between patients without AF history, with paroxysmal, and with persistent AF.

**Methods and results:**

Sixty consecutive patients with AF diagnosis (30 persistent and 30 paroxysmal) were enrolled and compared with 20 age-matched control; MDCT-derived images were post-processed to obtain 3D LA inFAT maps for all patients. Volume (mL) and mean signal intensities [(Hounsfield Units (HU)] of inFAT (HU −194; −5), dense inFAT (HU −194; −50), and fat-myocardial admixture (HU −50; −5) were automatically computed by the software. inFAT volume was significantly different across the three groups (*P* = 0.009), with post-hoc pairwise comparisons showing a significant increase in inFAT volume in persistent AF compared to controls (*P* = 0.006). Dense inFAT retained a significant difference also after correcting for body mass index (*P* = 0.028). In addition, more negative inFAT radiodensity values were found in AF patients. Regional distribution analysis showed a significantly higher regional distribution of LA inFAT at left and right superior pulmonary vein antra in AF patients.

**Conclusion:**

Persistent forms of AF are associated with greater degree of LA intramyocardial adipose infiltration, independently of body mass index. Compared to controls, AF patients present higher LA inFAT volume at left and right superior pulmonary vein antra.

## Graphical Abstract

**Graphical abstract:**
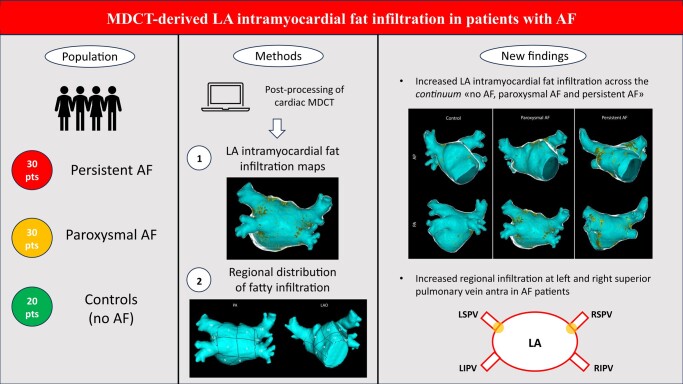
AF, atrial fibrillation; LA, left atrium; LSPV, left superior pulmonary vein; LIPV, left inferior pulmonary vein; MDCT, multi-detector computed tomography; RSPV, right superior pulmonary vein; RIPV, right inferior pulmonary vein

**What’s new?**

- Post-processing of multi-detector computed tomography images allows to create patient-specific three-dimensional left atrial myocardial fat infiltration maps;
- In non-excessively remodelled left atria, a greater degree of adipose infiltration at the level of atrial myocardium is associated with persistent forms of AF, independently of body mass index;
- Atrial fibrillation patients show a significantly higher relative infiltration of the proximal portion (antrum) of the two superior pulmonary veins, compared to controls.

## Introduction

Over the last two decades, a substantial amount of scientific evidence has been gathered, pointing towards a correlation between obesity and cardiac arrhythmias.^1^ In particular, obesity has emerged as an independent risk factor for the development of atrial fibrillation (AF), the most common arrhythmia encountered in clinical practice,^2–5^ with nearly an additional 25% risk of incident AF for every five-unit increase in body mass index (BMI).^6^ In addition, excessive body weight is also related to sub-optimal results of AF catheter ablation and weight loss has been proven to be beneficial, in a dose-dependent fashion, to increase the likelihood of long-term sinus rhythm maintenance after the intervention, as well as in reducing AF burden and symptoms severity in the general AF population.^2,7–10^

The mechanisms by which overweight and obesity contribute to the risk, progression, and severity of AF are multifactorial. In recent years, there has been increasing interest in the role of cardiac fat in the development of AF, particularly epicardial adipose tissue (EAT), which has been shown to be more prominent in patients with higher BMI.^11,12^ Several studies employing cardiac imaging techniques [primarily multi-detector computed tomography (MDCT)] have demonstrated an association between EAT and AF, with the former being an independent predictor of AF development and recurrence after catheter ablation.^13,14^ In addition, it was demonstrated a positive linear relationship between the increase of EAT and the continuum of no AF, paroxysmal AF, persistent AF, and long-lasting persistent AF.^15^ Several mechanisms have been proposed to explain how EAT might contribute to AF, including pro-inflammatory/pro-fibrotic paracrine influence on the underlying atrial myocardium, an increased activity of the ganglionated plexi (which are located inside EAT), and fatty infiltration of atrial myocardium (potentially altering local conduction and cellular electrophysiological properties).^16^ However, the role of this last potential mechanism [infiltrated adipose tissue (inFAT)] has been poorly investigated and all past research efforts have basically focused on EAT, most likely due to the difficulty in defining the epicardial aspect of the atrial myocardium by a standardized and reproducible approach.

We recently demonstrated that pre-procedural MDCT-derived images can be post-processed and the endocardial and epicardial aspects of the atrial myocardium can be easily and reproducibly segmented,^17^ with the potential to derive 3D left atrial wall thickness (LAWT) maps which can be used for a personalized AF ablation approach.^18,19^ Aim of the present study is to evaluate the feasibility of creating 3D LA inFAT maps and to compare the extent of inFAT between patients without AF history, with paroxysmal, and with persistent AF.

## Methods

### Patient sample

This study is a single-centre, observational, retrospective, proof-of-concept, case-control study. Consecutive patients >18 years old, referred to Teknon Medical Center (Barcelona, Spain) to undergo first AF ablation^20,21^ from January 2022 to February 2023, were screened for possible inclusion in the study. We excluded patients with significant LA remodelling (LA diameter ≥42 mm) in order to ensure greater comparability with a control group of non-AF patients; indeed, this cut-off has been clinically associated in previous studies with an increased risk of AF recurrence after catheter ablation.^22,23^ After applying the main exclusion criteria, 30 persistent AF patients and 30 propensity score-matched paroxysmal AF patients (based on age, sex, and LA diameter) were included. Paroxysmal and persistent AF was defined according to the definition of the last European Society of Cardiology (ESC) guidelines.^24^ The control group was constituted of 20 age-matched patients, without a history of AF, older than 18 years old, who underwent cardiac MDCT in the same time period.

The study complied with the Declaration of Helsinki and was approved by the Institutional Ethics Committee. All participants included in the study provided informed written consent.

### Pre-procedural multi-detector cardiac tomography and image post-processing

The use of pre-procedural cardiac imaging is increasingly supported by scientific evidence for accurate diagnostic classification, prognostic stratification, and peri-procedural support.^25–28^ In all patients, a pre-procedural MDCT was obtained with a Revolution™ CT scanner (General Electric Healthcare). The images were acquired during an inspiratory breath-hold using retrospective ECG-gating technique with tube current modulation set between 50% and 100% of the cardiac cycle. MDCT images were analysed with ADAS 3D™ software (ADAS3D Medical, Barcelona, Spain) to obtain 3D LAWT maps and 3D inFAT maps. Image post-processing was performed blinded to the allocation in the different study groups. LA endocardial layer was delineated by means of a semi-automatic using a threshold-based segmentation, while the epicardial layer was defined in a semi-automatic way by using an artificial-intelligence-based segmentation pipeline integrated into the software, which could be then manually re-adjusted by the user (in the present case, minor manual corrections were required in nearly all the patients). The reproducibility agreement of LAWT-maps derived by the described semi-automatic threshold-based segmentation was recently analysed, with the results of the studies reporting a colour agreement between LAWT maps of 95.5% and 92.9% intra- and inter-observer, respectively. For all analyses, the concordance increased with user-experience. Altogether, these results suggest that LAWT measurements are reproducible.^17^ Subsequently, LAWT was automatically computed at each point as the distance between each endocardial point and its projection to the epicardial shell and displayed as a colour-coded 3D map (red < 1 mm, 1 mm ≤ yellow < 2 mm, 2 mm ≤ green < 3 mm, 3 mm ≤ blue < 4 mm, and purple ≥ 4 mm). 3D inFAT maps were generated using a threshold-based segmentation of the volume between the endocardial and the epicardial LA shells. inFAT was defined as a tissue with reduced radiodensity, in the range of −194 Hounsfield Units (HU) to −5HU.^29,30^ In addition, two specific subranges were explored, the former between −194HU and −50HU (dense inFAT) and the latter between −50HU and −5HU (fat-myocardium admixture), as previously done by Sung *et al*.^29,30^ Left atrial appendage as well as the more distal segments of the pulmonary veins (>5 mm from the ostia) were excluded from the segmentation. Volume (mL) and mean signal intensities (HU) of inFAT, dense inFAT, and fat-myocardial admixture were automatically calculated by the software, as well as the LA volume (mL). Finally, we also computed inFAT, dense inFAT, and fat-myocardial admixture normalized values [arbitrary units—a.u.] by diving the original values by MDCT-derived patient-specific LA volume and by the segmented LA wall volume. This allowed us to conduct a sensitivity analysis, in addition to the comparison of the main outcome variables (absolute values of inFAT, dense inFAT, and fat-myocardial admixture), on volume-normalized metrics of inFAT and its subcomponents.

### Regional analysis of left atrial intramyocardial fat infiltration

**Figure 1:**
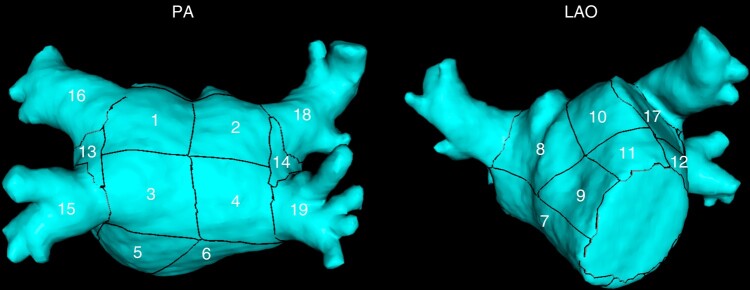
Nineteen-segment semi-automatic LA region segmentation. PA, postero-anterior projection; LAO, left anterior oblique projection; LA, left atrial.

Using a methodology that semi-automatically divides the LA into anatomically meaningful regions, we were able to assess the regional distribution of inFAT within the endocardium and the epicardium of the 3D LA shell. We adopted a modified version of the semi-automatic LA regionalization previously described by Benito *et al*.,^31^ which finally provides 19 LA segments (after excluding LA appendage and mitral valve), as depicted in *Figure 1*:

- Segments 1–4, posterior wall: these segments are bounded by the line between the superior edge of the ostium of superior pulmonary veins (PVs), the line that joins the inferior and superior ostia of homolateral PVs, and the line that joins the inferior edge of inferior PVs;
- Segments 5–6, floor: these segments are bounded by the inferior aspect of the posterior wall and the posterior aspect of the mitral annulus;
- Segment 7, interatrial septal wall;
- Segments 8–11, anterior wall: these segments are delimited by the superior aspect of the posterior wall and the anterior aspect of the mitral annulus and the LA appendage;
- Segment 12, left lateral wall: this segment is bounded by the anterior wall and the left floor (Segment 5) and it includes the mitral isthmus;
- Segment 13, left pulmonary veins carina;
- Segment 14, right pulmonary veins carina;
- Segment 15 and 16: left inferior pulmonary vein (LIPV) and left superior pulmonary vein (LSPV) antra, respectively;
- Segment 17: LA ridge;
- Segments 18 and 19: right inferior pulmonary vein (RIPV) and right superior pulmonary vein (RSPV) antra, respectively.

Using a custom-made MATLAB script, we evaluated segment-specific volumes of inFAT and we obtained and compared between the study sub-groups the regional distribution of left intra-atrial fat infiltration by calculating relative percentage of inFAT volume in every LA segment, defined as amount of fat in that region divided by the total amount of inFAT in LA wall. To put the regional inFAT volume in perspective, we also calculated the regional volume percentage, defined as the volume of a specific segment divided by the total segmented wall volume. This approach has been recently used to assess the regional distribution of MRI-based late gadolinium enhancement (LGE) by Assaf *et al*.^32^ in a post-hoc analysis of the DECAAF-II trial.

### Statistical analysis

In case of normal distribution, continuous variables were reported as mean ± standard deviation and were compared between different groups using two-sample *t*-test (two groups) or one-way analysis of variance (ANOVA) test (more than two groups; post-hoc *t*-test with Bonferroni correction used for pairwise sub-group comparison). If not normally distributed, continuous variables were reported as median with interquartile range (IQR) and between-group comparison was performed using Mann–Whitney *U* test (two groups) or Kruskall–Wallis test (more than two groups; post-hoc Kruskall–Wallis test with Bonferroni correction were used for pairwise sub-group comparison). Categorical variables were presented as counts and percentage, and between group comparison was performed using chi-squared test. Analysis of covariance (ANCOVA) was used to compare continuous outcome variables, adjusting for potential confounders. All statistical tests were two-tailed, and a significance level of *P* < 0.05 was used to determine statistical significance. Data were analysed with R version 4.0.0 (R Foundation for Statistical Computing, Vienna, Austria) and Matlab statistics toolbox (Matlab R2010a, The Mathworks, Inc., Natick, MA, USA).

## Results

*Table 1* reports the clinical characteristics of the 80 patients included in the present analysis, stratified by the study sub-groups. Overall, the majority of the patients were male (45, 56.2%), with a mean age of 64 ± 9.9 years and a mean LA diameter of 36.2 ± 3.1 mm. BMI was the only baseline clinical feature that differed significantly between the study groups (paroxysmal AF: 26.7 ± 3.9 kg/m^2^; persistent AF: 27.8 ± 3.9 kg/m^2^; controls: 24.8 ± 3.3 kg/m^2^; *P* = 0.028).

**Table 1:** Baseline characteristics of the included patients

	*Total (N* *=* *80)*	Paroxysmal AF (*N* = 30)	Persistent AF (*N* = 30)	Controls (*N* = 20)	*P*-value^a^
Sex					0.577
Male	45 (56.2%)	15 (50.0%)	17 (56.7%)	13 (65.0%)	
Female	35 (43.8%)	15 (50.0%)	13 (43.3%)	7 (35.0%)	
Age	64.0 ± 9.9	64.9 ± 10.2	64.1 ± 10.4	62.7 ± 9.0	0.737
BMI	26.7 ± 3.9	26.7 ± 3.9	27.8 ± 3.9	24.8 ± 3.3	**0**.**028**
Hypertension	32 (40.0%)	10 (33.3%)	16 (53.3%)	6 (30.0%)	0.164
Dyslipidemia	16 (20.0%)	8 (26.7%)	5 (16.7%)	3 (15.0%)	0.508
Diabetes	1 (1.2%)	1 (3.3%)	0 (0.0%)	0 (0.0%)	0.430
Smoker	3 (3.8%)	2 (6.7%)	1 (3.3%)	0 (0.0%)	0.472
CHA_2_DS_2_-VASc score	1.8 ± 1.4	1.7 ± 1.4	1.9 ± 1.5	1.2 ± 1^b^	0.151
LA diameter	36.2 ± 3.1	36.2 ± 2.9	36.9 ± 3.0	35.2 ± 3.2	0.149
LVEF	60.9 ± 5.3	61.8 ± 6.3	59.9 ± 5.9	60.6 ± 2.3	0.479

^a^Reported *P*-value refers to the comparison between the three sub-group of interest (chi-squared test, one-way ANOVA test and Kruskall–Wallis test, as appropriate); *P*-values < 0.05 are reported in bold.

^b^‘Potential’ CHA_2_DS_2_-VASc score (control patients are not AF patients).

AF, atrial fibrillation; LA, left atrial; LVEF, left ventricular ejection fraction.

MDCT images were post-processed, as detailed in the Methods section, to obtain 3D LAWT maps and 3D inFAT maps. The derived mean LAWT and LA volume were different across the three study groups (*Table 2*), with post-hoc analysis showing no significant differences between paroxysmal AF and persistent AF sub-group (*P* = 0.43 and 1.00 for LAWT and LA volume, respectively). Instead, we did not detect statistically significant differences in terms of LA segmented wall volume (*Table 2*).

**Table 2:** MDCT-derived LA volume, wall thickness and segmented wall volume, stratified by study sub-groups

	Total (*N* = 80)	Paroxysmal AF (*N* = 30)	Persistent AF (*N* = 30)	Controls (*N* = 20)	*P*-value^a^
LA volume (mL)	100 ± 20	106 ± 21	102 ± 14	89 ± 22	**0**.**007**
LA wall thickness (mm)	1.25 ± 0.22	1.25 ± 0.22	1.33 ± 0.20	1.13 ± 0.21	**0**.**006**
LA segmented wall volume (mL)	11.1 ± 4.1	11.3 ± 4.6	11.9 ± 4.3	9.7 ± 2.7	0.181

^a^Reported *P*-value refers to one-way ANOVA test between the three sub-group of interest; P-values < 0.05 are reported in bold.

AF, atrial fibrillation; LA, left atrial.

**Figure 2:**
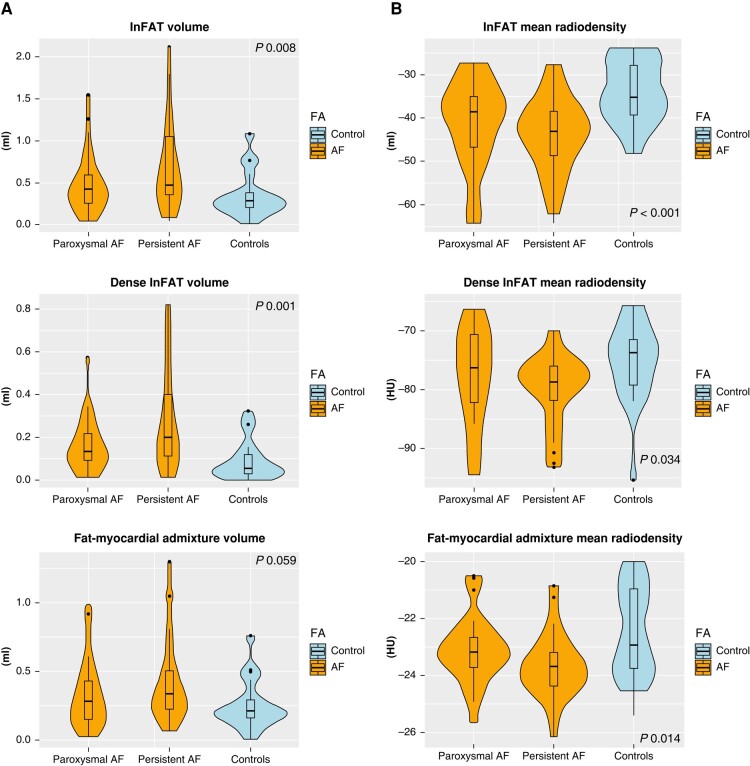
Violin plot reporting volumes and mean radiodensities of inFAT and its two subcomponents (dense inFAT and fat-myocardial admixture) in the study groups. *P*-values are referred to Kruskal–Wallis test. inFAT, infiltrated adipose tissue.

**Figure 3:**
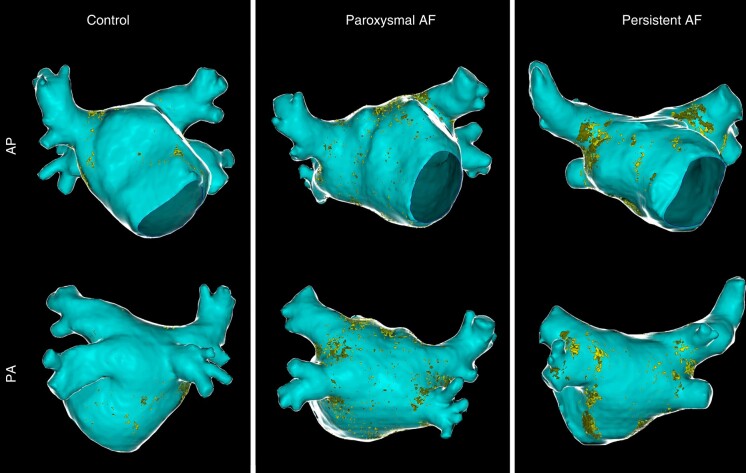
Antero-posterior (AP) and postero-anterior (PA) views of left intra-atrial fat maps in three patients from the different study groups (controls, paroxysmal AF, and persistent AF). Left atrial endocardial shell is depicted in light blue, while epicardial shell is shown in ‘glass-mode’. Dense fat and myocardial-fat admixture are reported in dark yellow and light yellow, respectively. AP, Antero-posterior; PA, postero-anterior; AF, atrial fibrillation.

**Figure 4:**
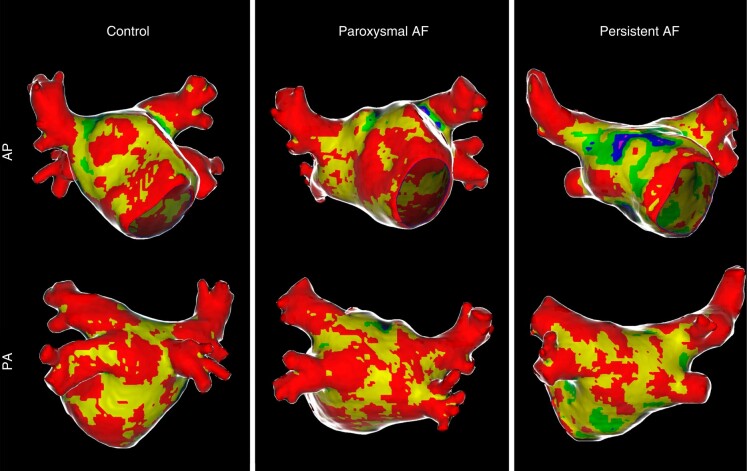
Antero-posterior (AP) and postero-anterior (PA) views of LAWT maps in the same three patients of the previous figure, from the different study groups (controls, paroxysmal AF, and persistent AF). AP, Antero-posterior; PA, postero-anterior; AF, atrial fibrillation; LAWT, left atrial wall thickness.

**Figure 5:**
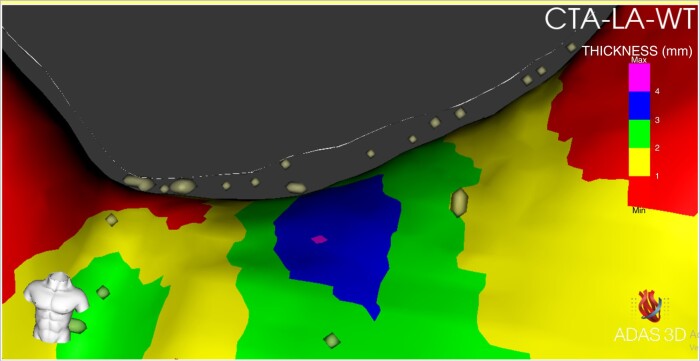
Zoomed antero-posterior (AP) view of the inFAT map, superimposed on the LA wall thickness map, from a paroxysmal AF patient showing that LA inFAT distribution was distributed throughout the atrial wall, with no preference for epicardial or endocardial location. CTA-LA-WT, computerized tomography angiography-derived left atrial wall thickness. AP, Antero-posterior; PA, postero-anterior; AF, atrial fibrillation, inFAT, infiltrated adipose tissue; LA, left atrial.

*Table 3* and *Figure 2* reports, respectively, median (IQR) and violin plot of inFAT, dense inFAT, and fat-myocardium admixture volumes, stratified by the study sub-groups. The median (IQR) HU of inFAT components is also reported in *Table 3*. InFAT volume was significantly different across the three study groups [persistent AF: 0.46 (0.35–1.45) mL; paroxysmal AF: 0.42 (0.25–0.59) mL; controls: 0.28 (0.20–0.38) mL; *P* = 0.009]. Post-hoc pairwise comparisons showed that persistent AF patients were characterized by an increased inFAT volume as compared to controls (*P* = 0.006), while other pairwise comparisons do not detect statistically significant differences. Concerning the two components of inFAT, dense inFAT volume was also significantly different between the study groups (*P* = 0.001), while fat-myocardium admixture volume showed a trend towards difference, albeit not formally reaching the statistical significance (*P* = 0.059). In the case of dense inFAT, the only significant difference detected at post-hoc pairwise comparisons was between persistent AF group and controls (*P* = 0.001). ANCOVA analysis revealed that, after correcting for the potential confounding factor of BMI, only dense inFAT volume retained a statistically significant difference across the three study sub-groups (*P* = 0.10, 0.028, and 0.27 for total inFAT, dense inFAT and fat-myocardial admixture, respectively). *Figure 3* shows an anterior–posterior and a posterior–anterior view of 3D LA inFAT maps for three paradigmatic cases, one per each study group, while *Figure 4* shows 3D LAWT maps for the same patients. LA inFAT was distributed throughout the atrial wall, with no preference for epicardial or endocardial location (*Figure 5*). The sensitivity analyses focusing on the volumes of inFAT and its subcomponents normalized by patient-specific MDCT-derived LA volume and by segmented LA wall volume showed that both the normalized metrics of dense inFAT were significantly different across the three sub-groups (*P* = 0.003 and 0.004, respectively). Also, total inFAT showed a statistically significant difference concerning LA volume-normalized values (*P* = 0.043), while the other normalized metrics were not significantly different across the three study groups (*Table 3*).

**Table 3:** Volume (mL), normalized volumes (a.u.) and mean intensity (HU) of InFAT and its subcomponents, stratified by study groups

	Total (*N* = 80)	Paroxysmal AF (*N* = 30)	Persistent AF (*N* = 30)	Controls (*N* = 20)	*P*-value^a^
Volumes (mL)					
InFAT	0.405 (0.245, 0.720)	0.415 (0.252, 0.593)	0.460 (0.350, 1.045)	0.275 (0.198, 0.375)	**0**.**008**
Dense InFAT	0.130 (0.057, 0.230)	0.130 (0.090, 0.215)	0.195 (0.110, 0.397)	0.050 (0.028, 0.118)	**0**.**001**
Fat-myocardial admixture	0.265 (0.170, 0.450)	0.280 (0.150, 0.432)	0.335 (0.233, 0.505)	0.210 (0.165, 0.290)	0.059
Normalized volumes (a.u.)					
InFAT (LA volume-normalized)	0.0040 (0.0024, 0.0066)	0.0038 (0.0022, 0.0060)	0.0052 (0.0031, 0.0096)	0.0035 (0.0019, 0.0056)	**0**.**043**
Dense InFAT (LA volume-normalized)	0.0012 (0.0006, 0.0022)	0.0012 (0.008, 0.0019)	0.0019 (0.0010, 0.0037)	0.0006 (0.0003, 0.0015)	**0**.**003**
Fat-myocardial admixture (LA volume-normalized)	0.0030 (0.0017, 0.0044)	0.0029 (0.0013, 0.0042)	0.0033 (0.0021,0.0054)	0.0025 (0.0017, 0.0041)	0.180
InFAT (segmented wall volume-normalized)	0.039 (0.024, 0.052)	0.040 (0.023, 0.049)	0.042 (0.030, 0.068)	0.034 (0.019, 0.042)	0.080
Dense InFAT (segmented wall volume-normalized)	0.011 (0.006, 0.017)	0.011 (0.007, 0.015)	0.015 (0.009, 0.029)	0.007 (0.003, 0.012)	**0**.**004**
Fat-myocardial admixture (segmented wall volume-normalized)	0.026 (0.017, 0.038)	0.026 (0.015, 0.034)	0.026 (0.020, 0.039)	0.023 (0.017, 0.033)	0.487
Mean intensity (HU)					
InFAT	−39.455 (−46.009, −34.636)	−38.621 (−46.639, −34.892)	−43.125 (−48.665, −38.430)	−34.862 (−39.281, −27.927)	**<0**.**001**
Dense InFAT	−77.145 (−80.847, −73.278)	−76.285 (−82.155, −70.593)	−78.665 (−81.755, −75.998)	−73.665 (−79.130, −71.382)	**0**.**034**
Fat-myocardial admixture	−23.465 (−24.000, −22.400)	−23.190 (−23.695, −22.667)	−23.710 (−24.378, −23.175)	−22.955 (−23.758, −20.975)	**0**.**014**

^a^Reported P-value refers to Kruskall–Wallis test between the three sub-group of interest; P-values < 0.05 are reported in bold.

AF, atrial fibrillation; LA, left atrial.

Concerning the median HU of inFAT and its two subcomponents (dense inFAT and fat-myocardium admixture), we found significant differences between the study groups (*P*-value <0.001, 0.034 and 0.014, respectively), with controls showing the least negative values [inFAT: −34.9 (−39.3; −27.9) HU; dense inFAT: −73.7 (−79.1; −71.4) HU; fat-myocardium admixture: −22.9 (−23.8; −21.0) HU] and persistent AF patients presenting the most negative ones [inFAT: −43.1 (−48.7; −38.4) HU; dense inFAT: −78.7 (−81.8; −76.0) HU; fat-myocardium admixture: −23.7 (−24.4; −23.2) HU]. The differences were statistically significant also adjusting for BMI (*P*-value <0.001, 0.027, and 0.004 for inFAT, dense inFAT, and fat-myocardial admixture, respectively).

**Figure 6:**
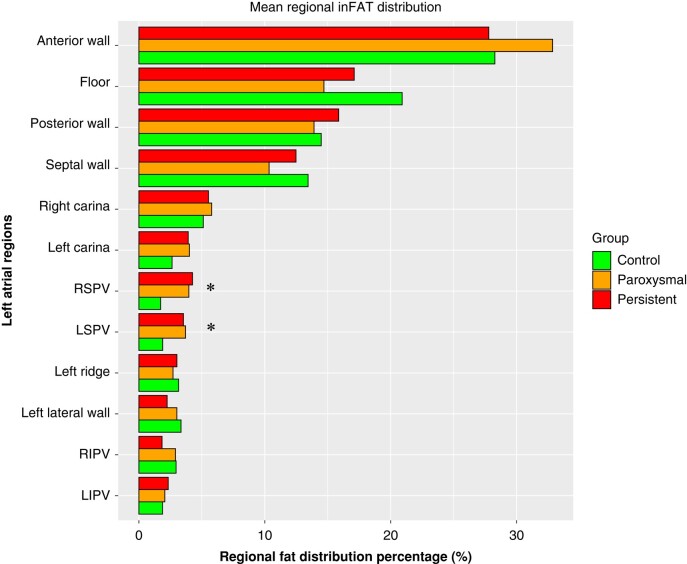
Regional inFAT distribution analysis, stratified by the three study groups. Bar plot reports, for each of the evaluated segment, the mean regional inFAT distribution percentage. The asterisk denotes statistically significant differences across the three study group (one-way ANOVA test). inFAT, infiltrated adipose tissue.

Finally, *Figure 6* shows the results of the regional assessment of fat infiltration, reporting the average segment-specific relative percentage of inFAT volume, stratified by each sub-group. A significant difference in the relative distribution of infiltrating fat was found for the left and right superior pulmonary vein antra [segment 16—LSPV (*P*-value 0.014), and segment 19—RSPV (*P*-value 0.002)], with AF patients presenting higher regional inFAT volume percentage in these segments. No statistically significant differences were found concerning segment-specific regional volume percentages (see Supplementary material online, *Table S1*).

## Discussion

In this study, performed in AF patients without a significantly remodelled LA and control patients without an history of AF, we found that by adequate post-processing of MDCT images, it is feasible to create patient-specific 3D fat infiltration maps of LA. Specifically, we found that the degree of LA fatty infiltration was different between the three study groups (paroxysmal AF, persistent AF, and control); of note, persistent AF patients showed the highest degree of LA fat infiltration, while control patients were characterized by the lowest inFAT amount. We have also observed a differential regional distribution of inFAT across the three study groups, with AF patients presenting higher relative percentage of inFAT localized at the antra of the superior pulmonary veins.

### inFAT volume and radiodensity

One of the possible ways in which EAT might contribute to AF is through fat infiltration of the underlying atrial myocardium.^16^ To date, however, no study have assessed *in vivo* the extent of myocardial fat infiltration at the level of LA. Based on recent demonstration that the epicardial aspect of the LA myocardium might be easily and reproducibly segmented through computerized tomography angiography (CTA) imaging,^17^ we here report, for the first time, that adequate post-processing of MDCT LA images allows to construct LA inFAT maps and to accurately quantify the degree of fat infiltration in the LA. Our data suggest that in patients who do not present a significantly remodelled left atrium (the study only included patients with a LA diameter >42 mm), the degree of LA fatty infiltration is associated with an increased probability of AF. Of note, the degree of fatty infiltration, at least in its dense subcomponent (which shows statistically significant difference both in absolute and in normalized values), is independent of BMI and it appears to be related to a gradient in the arrhythmic phenotype (from low level of fatty infiltration in control patients, to highest level of fatty infiltration in patients presenting with persistent form of AF). In addition, the analysis of the mean radiodensities of the inFAT gives additional relevant insights in the fatty infiltration process. In fact, we should take into account the phenomenon of the ‘partial volume effect’, which is the radiological phenomenon determines that, if a radiological pixel volume is comprised of a number of different substances (in this case, fat and atrial myocardium, considering that the infiltration might exist at an histological level and that the resolution of the CT is sub-millimetric), the resulting CT attenuation value (HU) represents some average of their properties. In this sense, the present finding of a gradient in the inFAT radiodensities across the study groups, which presented the most negative values in persistent AF patients and the least negative ones in control patients, supports the hypothesis of a higher fat-to-myocardial ratio in the evaluated voxels, adding further characterization of the fatty infiltration in addition to the inFAT volume analysis.

These results should be considered in the light of the findings of the elegant study recently published by Nalliah *et al*.^33^ In their work, the authors clearly demonstrated that infiltration of the atrial myocardium by EAT locally increased the conduction heterogeneity of the action potential, both by constituting a physical cellular barrier to the signal propagation and by a paracrine influence of the surrounding atrial myocytes, ultimately leading to an increased vulnerability of three-dimensional re-entrant circuits. This might help explaining why patients without the ‘classical’ substrate of an increased LA diameter might however present with a *de novo* persistent AF in case of significant LA fatty infiltration, which may constitute the predominant electrophysiological substrate in this kind of patients.

### inFAT regional distribution

The generation of the 3D fat infiltration maps also allows a detailed characterization of the regional distribution of the LA fatty infiltration. Overall, the superior sub-segments of the anterior wall (also including the LA roof) and septal wall showed the most prominent fatty infiltration. Focusing on potential difference in relative fat distribution across the three study groups, we found a significant difference in relative fat amount at the level of the superior pulmonary vein antra (RSPV and LSPV), with AF patients showing the highest relative percentage of fat infiltration in these sites and control patients the lowest values. Of note, considering that the mean amount of absolute fat volume in the overall LA was higher in persistent AF patients, this indicates that persistent AF forms present the highest magnitude of antral RSPV and LSPV fatty infiltration. This biological gradient might be suggestive of the fact that the total amount of inFAT is not the only relevant player in this process, but also the specific region of fatty infiltration might have an influence on the arrhythmic phenotype of the patients. In fact, it might be speculated that the present finding of an increased fatty infiltration at the level of the connexion site of the superior pulmonary veins and LA could constitute an element of further architectural complexity in an already peculiar anatomical site such as that of LA-pulmonary vein junction,^34^ potentially promoting anchoring of micro-re-entrant drivers that sustains more persistent forms of AF, even in relatively small LA. In addition, this finding could also imply a greater difficulty in performing transmural lesions at this level, due to the likely higher electrical impedance provided by the infiltrating fatty tissue,^35,36^ thus resulting in a worse long-term outcome of catheter ablation in such patients, whichever the energy source used.

### Limitations

Some limitations of our work must be acknowledged. First, small errors in technical and post-processing parameters may generate misinterpretations in the results, particularly related to the accuracy of manual and semi-automated identification of the endocardial and epicardial aspect of LA myocardium. However, as already stated, it was previously shown that with a minimum experience, the segmentation process was highly reproducible, thus lowering the likelihood of wrongly identifying the endo- and epicardial shell of LA myocardium. Second, the adopted HU range (−194 to −5 HU) and its two specific subranges (dense inFAT and fat-myocardial admixture) were originally used by Sung *et al*. in the ventricular myocardium^29,30^; slightly different cut-offs (−194 to −30 HU), without the distinctions in the two subranges, were used by Samanta *et al*. in the atrial myocardium,^37^ however, we used the cut-offs by Sung *et al*. because they provided the opportunity to better investigate the pathophysiology of the phenomenon, by separately assessing the dense fat infiltration and the fat-myocardial admixture component. We cannot, however, exclude that the presently used cut-off might be sub-optimal when applied to atrial myocardium. Third, the semi-automatic partitioning of LA in different segments, which originates from the previous experience of Benito *et al*.,^31^ might be an over-simplification of the different areas of LA, thus not being able to capture all the potential differences in regional inFAT distribution. Fourth, the findings of this present proof-of-concept study apply to a population with normal/mildly dilated LA and cannot be presently generalized to the whole AF population. Finally, even though the present findings support an association between LA intramyocardial fat infiltration and more persistent forms of AF, this does not prove a causal relationship.

## Conclusions

In non-excessively remodelled LA, a greater degree of adipose infiltration at the level of atrial myocardium is associated with persistent forms of AF, independently of BMI. In addition, the relative distribution of fat infiltration appears to be different between AF and control patients, with AF patients showing a significantly higher relative infiltration of the antrum of the two superior pulmonary veins.

## Supplementary Material

euad351_Supplementary_Data

## Data Availability

The data that support the findings of this study are available from the corresponding author, upon reasonable request.

## References

[1] Pabon MA, Manocha K, Cheung JW, Lo JC. Linking arrhythmias and adipocytes: insights, mechanisms, and future directions. Front Physiol 2018;9:1752. 30568603 10.3389/fphys.2018.01752 PMC6290087

[2] Lavie CJ, Pandey A, Lau DH, Alpert MA, Sanders P. Obesity and atrial fibrillation prevalence, pathogenesis, and prognosis: effects of weight loss and exercise. J Am Coll Cardiol 2017;70:2022–35. 29025560 10.1016/j.jacc.2017.09.002

[3] Dong XJ, Wang BB, Hou FF, Jiao Y, Li HW, Lv SP et al Global burden of atrial fibrillation/atrial flutter and its attributable risk factors from 1990 to 2019. Europace 2023;25:793–803. 36603845 10.1093/europace/euac237 PMC10062373

[4] Kalarus Z, Mairesse GH, Sokal A, Boriani G, Sredniawa B, Casado-Arroyo R et al Searching for atrial fibrillation: looking harder, looking longer, and in increasingly sophisticated ways. An EHRA position paper. Europace 2023;25:185–98. 36256580 10.1093/europace/euac144 PMC10112840

[5] Svennberg E, Tjong F, Goette A, Akoum N, Di BL, Bordachar P et al How to use digital devices to detect and manage arrhythmias: an EHRA practical guide. Europace 2022;24:979–1005. 35368065 10.1093/europace/euac038 PMC11636571

[6] Wong CX, Sullivan T, Sun MT, Mahajan R, Pathak RK, Middeldorp M et al Obesity and the risk of incident, post-operative, and post-ablation atrial fibrillation: a meta-analysis of 626,603 individuals in 51 studies. JACC Clin Electrophysiol 2015;1:139–52. 29759357 10.1016/j.jacep.2015.04.004

[7] Saglietto A, Gaita F, Blomstrom-Lundqvist C, Arbelo E, Dagres N, Brugada J et al AFA-Recur: an ESC EORP AFA-LT registry machine-learning web calculator predicting atrial fibrillation recurrence after ablation. Europace 2023;25:92–100. 36006664 10.1093/europace/euac145 PMC10103564

[8] Pathak RK, Middeldorp ME, Meredith M, Mehta AB, Mahajan R, Wong CX et al Long-term effect of goal-directed weight management in an atrial fibrillation cohort: a long-term follow-up study (LEGACY). J Am Coll Cardiol 2015;65:2159–69. 25792361 10.1016/j.jacc.2015.03.002

[9] Mulder MJ, Kemme MJB, Allaart CP. Radiofrequency ablation to achieve durable pulmonary vein isolation. Europace 2022;24:874–86. 34964469 10.1093/europace/euab279

[10] Lee G, Baker E, Collins R, Merino JL, Desteghe L, Heidbuchel H. The challenge of managing multimorbid atrial fibrillation: a pan-European European Heart Rhythm Association (EHRA) member survey of current management practices and clinical priorities. Europace 2022;24:2004–14. 36036694 10.1093/europace/euac136 PMC9733957

[11] Aitken-Buck HM, Moharram M, Babakr AA, Reijers R, Van HI, Fomison-Nurse IC et al Relationship between epicardial adipose tissue thickness and epicardial adipocyte size with increasing body mass index. Adipocyte 2019;8:412–20. 31829077 10.1080/21623945.2019.1701387 PMC6948959

[12] Poggi AL, Gaborit B, Schindler TH, Liberale L, Montecucco F, Carbone F. Epicardial fat and atrial fibrillation: the perils of atrial failure. Europace 2022;24:1201–12. 35274140 10.1093/europace/euac015

[13] Wong CX, Ganesan AN, Selvanayagam JB. Epicardial fat and atrial fibrillation: current evidence, potential mechanisms, clinical implications, and future directions. Eur Heart J 2017;38:1294–302. 26935271 10.1093/eurheartj/ehw045

[14] Jhuo SJ, Hsieh TJ, Tang WH, Tsai WC, Lee KT, Yen HW et al The association of the amounts of epicardial fat, P wave duration, and PR interval in electrocardiogram. J Electrocardiol 2018;51:645–51. 29997005 10.1016/j.jelectrocard.2018.04.009

[15] Wong CX, Abed HS, Molaee P, Nelson AJ, Brooks AG, Sharma G et al Pericardial fat is associated with atrial fibrillation severity and ablation outcome. J Am Coll Cardiol 2011;57:1745–51. 21511110 10.1016/j.jacc.2010.11.045

[16] Iacobellis G . Epicardial adipose tissue in contemporary cardiology. Nat Rev Cardiol 2022;19:593–606. 35296869 10.1038/s41569-022-00679-9 PMC8926097

[17] Valles-Colomer A, Rubio Forcada B, Soto-Iglesias D, Planes X, Trueba R, Teres C et al Reproducibility analysis of the computerized tomography angiography-derived left atrial wall thickness maps. J Interv Card Electrophysiol 2023;66:1045–55. 36802003 10.1007/s10840-023-01472-5

[18] Teres C, Soto-Iglesias D, Penela D, Jáuregui B, Ordoñez A, Chauca A et al Personalized paroxysmal atrial fibrillation ablation by tailoring ablation index to the left atrial wall thickness: the ‘ablate by-LAW’ single-centre study-a pilot study. Europace 2022;24:390–9. 34480548 10.1093/europace/euab216

[19] Falasconi G, Penela D, Soto-Iglesias D, Francia P, Teres C, Saglietto A et al Personalized pulmonary vein antrum isolation guided by left atrial wall thickness for persistent atrial fibrillation. Europace 2023;25:5–8. 10.1093/europace/euad118 PMC10228614 37125968

[20] Iliodromitis K, Lenarczyk R, Scherr D, Conte G, Farkowski MM, Marin F et al Patient selection, peri-procedural management, and ablation techniques for catheter ablation of atrial fibrillation: an EHRA survey. Europace 2023;25:667–75. 36512365 10.1093/europace/euac236 PMC9935016

[21] Fabritz L, Crijns HJGM, Guasch E, Goette A, Häusler KG, Kotecha D et al Dynamic risk assessment to improve quality of care in patients with atrial fibrillation: the 7th AFNET/EHRA consensus conference. Europace 2021;23:329–44. 33555020 10.1093/europace/euaa279 PMC13453135

[22] Liao YC, Liao JN, Lo LW, Lin YJ, Chang SL, Hu YF et al Left atrial size and left ventricular end-systolic dimension predict the progression of paroxysmal atrial fibrillation after catheter ablation. J Cardiovasc Electrophysiol 2017;28:23–30. 27779351 10.1111/jce.13115

[23] Kornej J, Hindricks G, Shoemaker MB, Husser D, Arya A, Sommer P et al The APPLE score: a novel and simple score for the prediction of rhythm outcomes after catheter ablation of atrial fibrillation. Clin Res Cardiol 2015;104:871–6. 25876528 10.1007/s00392-015-0856-x PMC4726453

[24] Hindricks G, Potpara T, Dagres N, Arbelo E, Bax JJ, Blomström-Lundqvist C et al 2020 ESC guidelines for the diagnosis and management of atrial fibrillation developed in collaboration with the European Association of Cardio-Thoracic Surgery (EACTS): the task force for the diagnosis and management of atrial fibrillation of the European Society of Cardiology (ESC) developed with the special contribution of the European Heart Rhythm Association (EHRA) of the ESC. Eur Heart J 2021;42:373–498. 32860505 10.1093/eurheartj/ehaa612

[25] Berruezo A, Penela D, Jáuregui B, de Asmundis C, Peretto G, Marrouche N et al Twenty-five years of research in cardiac imaging in electrophysiology procedures for atrial and ventricular arrhythmias. Europace 2023;25:euad183. 37622578 10.1093/europace/euad183 PMC10450789

[26] Francia P, Viveros D, Falasconi G, Soto-Iglesias D, Fernández-Armenta J, Penela D et al Computed tomography-based identification of ganglionated plexi to guide cardioneuroablation for vasovagal syncope. Europace 2023;6:5–8. 10.1093/europace/euad170 PMC10311406 37343139

[27] Jáuregui B, Soto-Iglesias D, Penela D, Acosta J, Fernández-Armenta J, Linhart M et al Cardiovascular magnetic resonance determinants of ventricular arrhythmic events after myocardial infarction. Europace 2022;24:938–47. 34849726 10.1093/europace/euab275

[28] Falasconi G, Penela D, Soto-Iglesias D, Jáuregui B, Chauca A, Antonio RS et al A standardized stepwise zero-fluoroscopy approach with transesophageal echocardiography guidance for atrial fibrillation ablation. J Interv Card Electrophysiol 2022;64:629–39. 34757547 10.1007/s10840-021-01086-9

[29] Sung E, Prakosa A, Aronis KN, Zhou S, Zimmerman SL, Tandri H et al Personalized digital-heart technology for ventricular tachycardia ablation targeting in hearts with infiltrating adiposity. Circ Arrhythm Electrophysiol 2020;13:E008912. 33198484 10.1161/CIRCEP.120.008912 PMC7738410

[30] Sung E, Prakosa A, Zhou S, Berger RD, Chrispin J, Nazarian S et al Fat infiltration in the infarcted heart as a paradigm for ventricular arrhythmias. Nat Cardiovasc Res 2022;1:933–45. 36589896 10.1038/s44161-022-00133-6 PMC9802586

[31] Benito EM, Cabanelas N, Nuñez-Garcia M, Alarcón F, Figueras I Ventura RM, Soto-Iglesias D et al Preferential regional distribution of atrial fibrosis in posterior wall around left inferior pulmonary vein as identified by late gadolinium enhancement cardiac magnetic resonance in patients with atrial fibrillation. Europace 2018;20:1959–65. 29860416 10.1093/europace/euy095

[32] Assaf A, Mekhael M, Noujaim C, Chouman N, Younes H, Feng H et al Effect of fibrosis regionality on atrial fibrillation recurrence: insights from DECAAF II. Europace 2023;25:euad199. 37428891 10.1093/europace/euad199 PMC10519620

[33] Nalliah CJ, Bell JR, Raaijmakers AJA, Waddell HM, Wells SP, Bernasochi GB et al Epicardial adipose tissue accumulation confers atrial conduction abnormality. J Am Coll Cardiol 2020;76:1197–211. 32883413 10.1016/j.jacc.2020.07.017

[34] Ho SY, Cabrera JA, Tran VH, Farré J, Anderson RH, Sánchez-Quintana D. Architecture of the pulmonary veins: relevance to radiofrequency ablation. Heart 2001;86:265–70. 11514476 10.1136/heart.86.3.265 PMC1729909

[35] Barkagan M, Rottmann M, Leshem E, Shen C, Buxton AE, Anter E. Effect of baseline impedance on ablation lesion dimensions: a multimodality concept validation from physics to clinical experience. Circ Arrhythm Electrophysiol 2018;11:e006690. 30354405 10.1161/CIRCEP.118.006690

[36] Nattel S, Aguilar M. Electrophysiological effects of atrial epicardial adipose tissue: keep your friends close and your enemies closer. J Am Coll Cardiol 2020;76:1212–4. 32883414 10.1016/j.jacc.2020.07.031

[37] Samanta R, Houbois CP, Massin SZ, Seidman M, Wintersperger BJ, Chauhan VS. Interatrial septal fat contributes to interatrial conduction delay and atrial fibrillation recurrence following ablation. Circ Arrhythm Electrophysiol 2021;14:e010235. 34583515 10.1161/CIRCEP.121.010235

