# Supplementary material for: Assessing left atrial intramyocardial fat infiltration from computerized tomography angiography in patients with atrial fibrillation
Source: Europace. 2023 Nov 27;25(12):euad351. doi: 10.1093/europace/euad351 (PMC10751854; doi:10.1093/europace/euad351)
Supplement: euad351_Supplementary_Data [file euad351_supplementary_data.docx]

**Assessing left atrial intramyocardial fat infiltration from computerized tomography angiography in patients with atrial fibrillation**

*Supplementary Material*

**Supplementary Table 1. Regional volume percentage, stratified by the study subgroup.**

|  | Total (N=80) | Control (N=20) | Paroxysmal AF (N=30) | Persistent AF (N=30) | p value* |
| --- | --- | --- | --- | --- | --- |
| **Interatrial septal wall** |  |  |  |  | 0.621 |
| Mean (SD) | 12.5 (3.2) | 12.7 (2.9) | 12.0 (3.3) | 12.8 (3.3) |  |
| Range | 5.8 - 20.8 | 8.3 - 19.0 | 7.4 - 20.7 | 5.8 - 20.8 |  |
| **Left lateral wall** |  |  |  |  | 0.375 |
| Mean (SD) | 3.8 (2.2) | 3.6 (2.2) | 4.3 (2.1) | 3.5 (2.3) |  |
| Range | 0.1 - 10.8 | 0.4 - 7.4 | 0.5 - 8.8 | 0.1 - 10.8 |  |
| **Left pulmonary veins carina** |  |  |  |  | 0.319 |
| Mean (SD) | 2.4 (1.1) | 2.1 (1.2) | 2.6 (1.1) | 2.4 (1.0) |  |
| Range | 0.4 - 5.7 | 0.4 - 5.7 | 1.0 - 5.0 | 0.9 - 4.4 |  |
| **Right pulmonary veins carina** |  |  |  |  | 0.346 |
| Mean (SD) | 2.5 (1.0) | 2.3 (0.9) | 2.5 (0.8) | 2.7 (1.3) |  |
| Range | 0.3 - 5.8 | 0.3 - 3.8 | 1.0 - 3.9 | 0.8 - 5.8 |  |
| **Left inferior pulmonary vein (LIPV)** |  |  |  |  | 0.443 |
| Mean (SD) | 1.0 (0.4) | 1.0 (0.6) | 1.1 (0.4) | 0.9 (0.3) |  |
| Range | 0.3 - 2.9 | 0.3 - 2.9 | 0.5 - 2.0 | 0.5 - 2.0 |  |
| **Left superior pulmonary vein (LSPV)** |  |  |  |  | 0.051 |
| Mean (SD) | 1.5 (0.6) | 1.4 (0.4) | 1.7 (0.7) | 1.3 (0.4) |  |
| Range | 0.5 - 4.6 | 0.7 - 2.3 | 0.9 - 4.6 | 0.5 - 1.8 |  |
| **Left atrial ridge** |  |  |  |  | 0.642 |
| Mean (SD) | 5.7 (3.1) | 5.4 (2.6) | 5.5 (2.9) | 6.2 (3.5) |  |
| Range | 2.0 - 16.3 | 2.4 - 12.1 | 2.0 - 13.9 | 2.3 - 16.3 |  |
| **Right inferior pulmonary vein (RIPV)** |  |  |  |  | 0.257 |
| Mean (SD) | 0.8 (0.3) | 0.8 (0.4) | 0.8 (0.3) | 0.7 (0.2) |  |
| Range | 0.2 - 1.9 | 0.2 - 1.9 | 0.3 - 1.5 | 0.2 - 1.2 |  |
| **Right superior pulmonary vein (RSPV)** |  |  |  |  | 0.182 |
| Mean (SD) | 1.5 (0.6) | 1.3 (0.6) | 1.6 (0.6) | 1.4 (0.5) |  |
| Range | 0.6 - 3.3 | 0.6 - 3.1 | 0.7 - 3.3 | 0.8 - 2.7 |  |
| **Posterior wall** |  |  |  |  | 0.739 |
| Mean (SD) | 16.9 (4.0) | 16.5 (3.5) | 16.8 (4.5) | 17.4 (4.0) |  |
| Range | 8.0 - 27.5 | 10.5 - 25.7 | 8.0 - 24.7 | 10.1 - 27.5 |  |
| **Floor** |  |  |  |  | 0.258 |
| Mean (SD) | 20.0 (4.0) | 21.2 (4.0) | 19.2 (3.8) | 19.9 (4.3) |  |
| Range | 10.6 - 28.5 | 14.2 - 27.5 | 12.6 - 26.6 | 10.6 - 28.5 |  |
| **Anterior wall** |  |  |  |  | 0.618 |
| Mean (SD) | 31.4 (4.4) | 31.6 (4.3) | 31.8 (4.8) | 30.7 (4.0) |  |
| Range | 22.3 - 40.1 | 23.7 - 40.1 | 22.4 - 39.9 | 22.3 - 38.8 |  |

*reported p-value refers to one-way ANOVA test between the three subgroup of interest
